# Combining local and global cues to motion

**DOI:** 10.3758/s13414-017-1380-z

**Published:** 2017-09-06

**Authors:** Michael Morgan

**Affiliations:** Division of Optometry, School of Health Sciences, City, University of London, Northampton Square, London, EC1V0HB UK

**Keywords:** Motion perception, Double-drift, Fraser twisted cord, Global intergration

## Abstract

A spinning, moving object, such as a football with a surface texture, combines motion signals from rotation and translation. The interaction between these two kinds of signal was studied psychophysically with moving, circular clouds of dots, which also could move within the cloud. If the cloud moved near-vertically downwards but the dots within it moved obliquely, the apparent path of the cloud was attracted to that of the dots, as previously demonstrated with moving Gabor patches (Tse & Hseih *Vision Research, 46*, 3881-3885, 2006; Lisi & Cavanagh *Current Biology, 25*, 2535-40, 2015). This attractive effect was enhanced in parafoveal viewing and by not presenting a frame around the dots. A larger effect in the opposite direction (repulsion) was found for the perceived direction of the dots when they moved near-vertically and the cloud containing them moved obliquely. These results are discussed in relation to Gestalt principles of perceived relative motion and, more recently, Bayes-inspired accounts of the interaction between local and global motion.

## Introduction

**Fig. 1:**
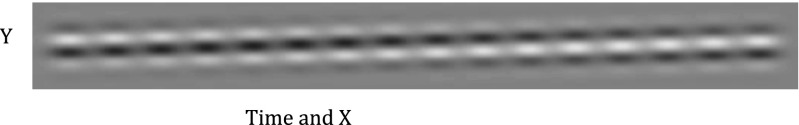
Composite space-time diagram of a Gabor stimulus moving from left to right with its carrier grating moving upwards

Several well-known demonstrations by the Gestalt school showed that it is difficult for observers to sense the trajectory of a moving object in retinal coordinates when it is accompanied by other objects, such as moving frame (Dunkner, 1938). Johansson (1975) showed that the perceived trajectory of a moving dot could be profoundly altered by that of flanking dots. More recently, interest in the principles of motion integration has resurfaced in the form of the “infinite regress” or “double drift” stimulus (Tse, & Hsieh, 2006; Kwon, Tadin & Knill, 2015; Lisi & Cavanagh, 2015). In one version of the stimulus, a single Gabor patch moves in one direction while its carrier drifts in the orthogonal direction. Particularly in peripheral vision, the patch appears to move obliquely in the direction of the carrier, rather than in the direction of the envelope. The stimulus is shown in the Space-Time diagram in Fig. 1. If the carrier were moving upwards and the envelope rightwards, the perceived direction would be to the upper right.

Figure 1 also illustrates a classical spatial phenomenon: the “twisted cord” (Fraser, 1908; Morgan & Baldassi, 1997). The string of gratings considered as single object appears to tilt upwards and to the right. It also has been shown that the “twisted cord” has a reciprocal effect: the apparent orientation of the carrier grating is attracted to that of an elongated, tilted envelope (Morgan, Mason & Baldassi, 2000). The purpose of the experiments reported was to investigate whether there also is a reciprocal effect in the motion domain. Instead of gratings, we used a moving frame containing moving dots. This avoids the inherent ambiguity of the motion of a one-dimensional grating within an aperture (Wallach, 1935; Wuerger, Shapley and Rubin, 1996).

## Methods

Experiments were conducted in a specially constructed light-proof room with matte-black surfaces to minimize reflections from the monitor. The latter was the only source of illumination. Its boundaries were clearly visible providing a reference for the vertical, which was the same as the gravitationally vertical. Stimuli were presented on a 60-Hz frame-rate Sony Trinitron CRT monitor, viewed from 75 cm so that 1 pixel subtended 1.275 arcmin at the observer’s eye (Morgan, Schreiber & Solomon, 2016).

**Fig. 2:**
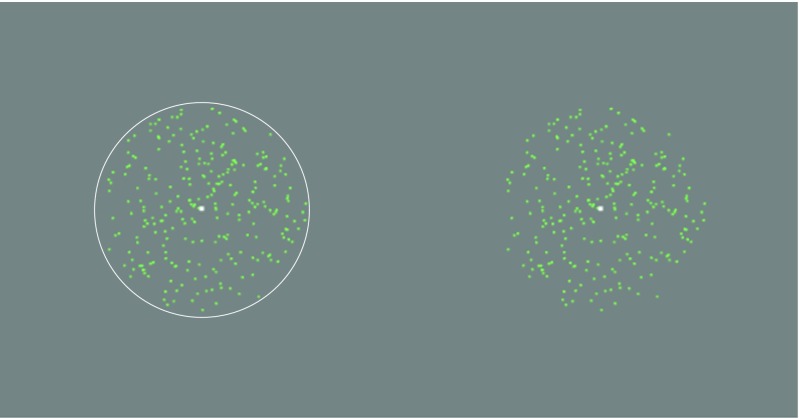
Illustrations of stimuli similar to those used in the experiments. The green dots moved near-vertically down the screen. In Experiments 1 & 2 (left) the dots were visible only within a circular frame (the envelope), which also moved downwards but not necessarily with the same angle as the dots. In Experiment 3 (right), the envelope was not explicitly marked with a circle. The white fixation point was stationary throughout stimulus presentation. Two stimuli (the “probes”) were presented in succession and the observer had to decide in which of them the dots moved more vertically (Experiment 1) or in which of them the envelope moved more vertically (Experiments 2 and 3)

The basic stimulus consisted of a cloud of dots presented on the monitor (Fig. 2). The dot cloud was generated initially to cover the whole screen but was made visible only within a circular aperture (hereafter called the “envelope”). The diameter of the circular envelope was ~2.5 °; the dot diameter was 0.0425°; in Experiments 1 and 2 the dot lifetime was 5 refreshes (80 ms); in Experiment 3 it was as long as the presentation. Dots (512) were generated in random positions over a larger area than the envelope but were only visible within the envelope, and fell within the envelope randomly such that the expected value of their number was 60. In Experiments 1 and 2, the cloud was surrounded by a white circular frame. In Experiment 3, the frame was absent. The luminances of the background, the dots, and the circular frame were 23, 75, and 92 cd/m^2^ respectively. The speed of the circular frame was 6.37 deg/s, and its excursion from start to finish was 5.578 deg. The centre of the trajectory was always the centre of the screen, but the fixation point was randomly jittered from the centre in the horizontal direction over presentations by random sampling from a rectangular PDF with width 1.275 deg. The jitter was independent in the two intervals of the 2AFC to avoid the use of alignment cues. In Experiment 3, the fixation point was displaced from the centre of the trajectory by 4.25 deg so that the stimulus appeared in the parafovea.

A 2AFC procedure was used to reduce the plausibility of non-perceptual biases masquerading as perceptual biases (Morgan, Melmoth, & Solomon, 2013). On each trial, two dot-clouds (the “probes”) were presented in succession. In Experiment 1, observers were asked which dot cloud contained dots that moved more vertically (i.e., less obliquely), with respect to the screen. They were explicitly asked to ignore envelope motion. In Experiments 2 and 3, they were asked which envelope moved more vertically, with respect to the screen. In these experiments, observers were explicitly asked to ignore dot motion.

The two probes were defined by the angles that their relevant components (i.e., dots in Experiment 1, envelopes in Experiments 2 and 3) formed with the vertical meridian. One of them, the standard, had an angle we refer to as the pedestal. The other probe had an angle that was the sum of the same pedestal and another angle called the test. Probe sequence (i.e., standard first or standard second) was determined randomly (with replacement) on each trial. The combination of pedestal and test also was determined randomly, but with replacement. Pedestal angles *p* (in degrees) were selected from the set {−5, 0, 5}. Test angles *t* (also in degrees) were selected from the set {−8, −6, −4, −2, 2, 4, 6, 8}.

For half of the trials in Experiment 1, the envelope angle A_e_ was +30 deg to vertical; for the other half, it was −30 deg and similarly for the dot angle A_d_ in Experiment 2. There were four trials per session for each combination of pedestal, test, and envelope/dot angle, making a total of 192 trials per session. Every 50 trials in the session, a message on the screen invited the observer to rest for as long as they wished.

**Fig. 3:**
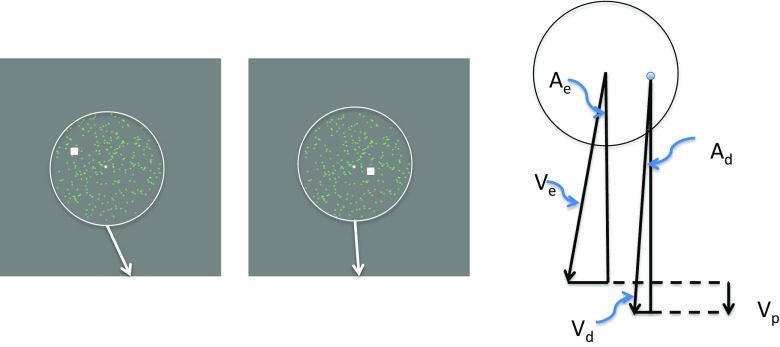
The two panels on the left show single frames from two successive presentations of the stimulus, which drifted downwards from a position above the fixation point (white square) to a position below, with the velocity vector of the envelope shown by the arrow. The observer’s task was to decide which of the two stimuli appeared to have the more vertical trajectory. The right-hand panel shows the velocity vectors of a circular frame and dots separately, along with their H and V components. *A* refers to the angle of the vector, *V* refers to its speed. Proper motion, V_p_ , is the vertical component of the dots’ velocity that is not shared by their envelope. Note the “jitter” of the fixation point position between presentations

Just as dot and envelope angles (A_d_ and A_e_ respectively) differed, dot and envelope velocities (V_d_ and V_e_ respectively) differed. The vertical component of the dots’ velocity exceeded that of their envelope’s velocity by a positive value V_p_, which we call “proper motion” (Fig. 3).

The participants were the author (MM), three experienced postdocs (KS, BD, NN), one experienced PhD student (JF), one naive paid volunteer (TP), and one naïve BSc student, carrying out a summer internship (MK). The subjects in Experiment 1 were MM, MK, KS, JF, TP, and BD; in Experiment 2, MM, MK, KS, JF, TP, and BD, and in Experiment 3 MM, NN, KS, JF, TP, and BD.

## Signal-detection model

We assume that observers base their decisions on two internal signals (generated by the two probes), each of which is corrupted by additive Gaussian noise, with variance *σ* ^2^/2. These internal signals vary linearly with the angle of the relevant stimulus component (i.e., dots in Experiment 1, envelopes in Experiments 2 and 3), but they may not be strictly proportional to that component. That is, they may have an additional offset from zero (*μ*) caused by the perceptual context, such as might be provided by effect of the envelope angle in Experiment 1. The observer decides which of these signals is more different from zero, and chooses appropriately. The purpose of the experiments is to determine the value of *μ*, the perceptual effect of context.

Data from each session were fit with a two-parameter signal-detection model, to obtain values (*μ*) and *σ*.

Within the context of signal-detection theory (Green & Swets, 1966), the internal signals can be described by normal distributions *S* and *T*, such that *S ~ N*(*p* + *μ*, *σ* ^2^/2) and *T ~ N*(*p* + *t* + *μ*, *σ* ^2^/2). Given these definitions, the probability of choosing the pedestal is given by

1 $$ {\displaystyle \begin{array}{c}\hfill \Pr \left({}^{"}{\mathrm{S}}^{"}\right)=\Pr \left(\left|S\right|<\left|T\right|\right)\hfill \\ {}\hfill =\Pr \left(\frac{S^2}{T^2}<1\right).\hfill \end{array}} $$

Morgan et al. (2015) noted that *S* ^2^/*T* ^2^ is a random variable having a doubly noncentral *F*-distribution. Its denominator's noncentrality parameter is 2(*p* + *μ* + *t*)^2^/*σ* ^2^, its numerator's noncentrality parameter is 2(*p* + *μ*)^2^/*σ* ^2^, and both denominator and numerator have 1 degree of freedom.

## Results

**Fig. 4:**
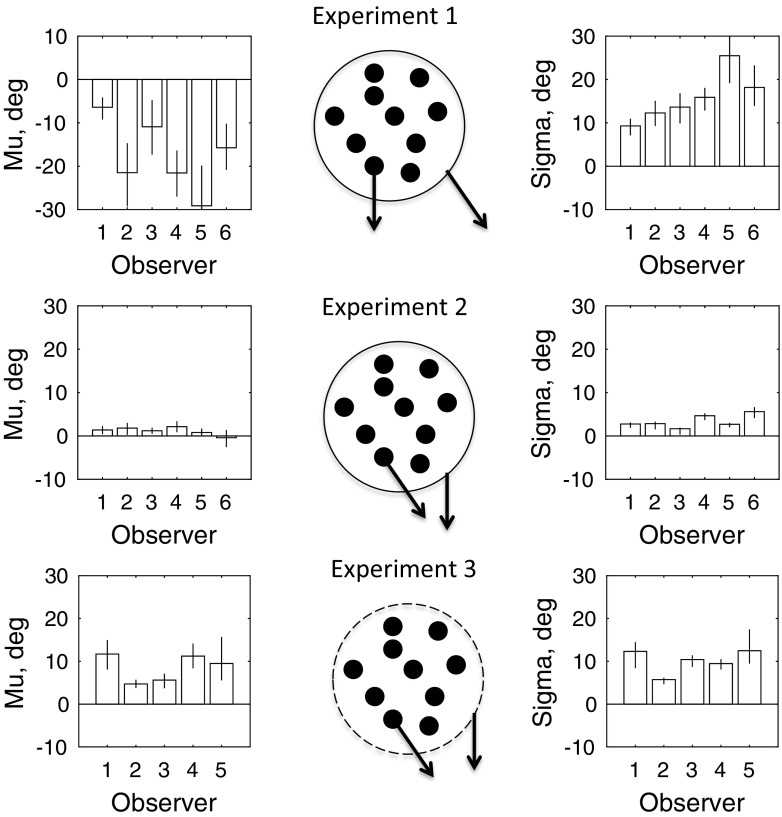
Results of Experiments 1-3. The schematics in the centre show the dots (filled circles) and an arrow, attached to one of the dots, denoting their direction. The dashed line circle in Experiment 3 shows the envelope, which was not actually presented on the screen, and an attached arrow indicating direction. The solid line envelopes in Experiments 1 and 2 were actually present on the screen. For further explanation see the text

The results of the three experiments are combined in Fig. 4. The bar graphs in the left side of the figure show the values of *μ* the perceptual shift in direction due to context. Negative values indicate repulsion of the perceived dot direction from that of the envelope (Experiment 1). Positive values indicate attraction of the perceived direction of the envelope to that of the dots (Experiment 2 and 3). The right side of the figure shows the corresponding values of *σ*, the internal noise. The repulsion of the dot trajectories from that of the envelope in Experiment 1 was significantly different from zero (*μ* = −17.52 deg; *t* = 5.23; df = 5; *p* = 0.0034). The attraction of the perceived envelope angle to that of the dots in Experiment 2 was much smaller but still significant (*μ* = 1.16 deg; *t* = 3.12; df = 5; *p* = 0.0262). The attraction in Experiment 3 was larger and also significant (*μ* = 8.42 deg; *t* = 5.63, df = 4; *p* = 0.0049). The small effect found in Experiment 2 is different from the much larger effects in the “infinite regress” stimulus reported by others (Tse, & Hsieh, 2006; Kwon et al., 2015; Lisi & Cavanagh, 2015). However, these previous studies did not use a hard aperture like our circle and were performed with eccentric fixation; nor was an equivalent of our limited lifetime used, because the experiments employed gratings. In Experiment 3, we used eccentric fixation, no aperture, and dot lifetime equal to the duration of the stimulus. This increased the magnitude of the perceptual offset.

**Fig. 5:**
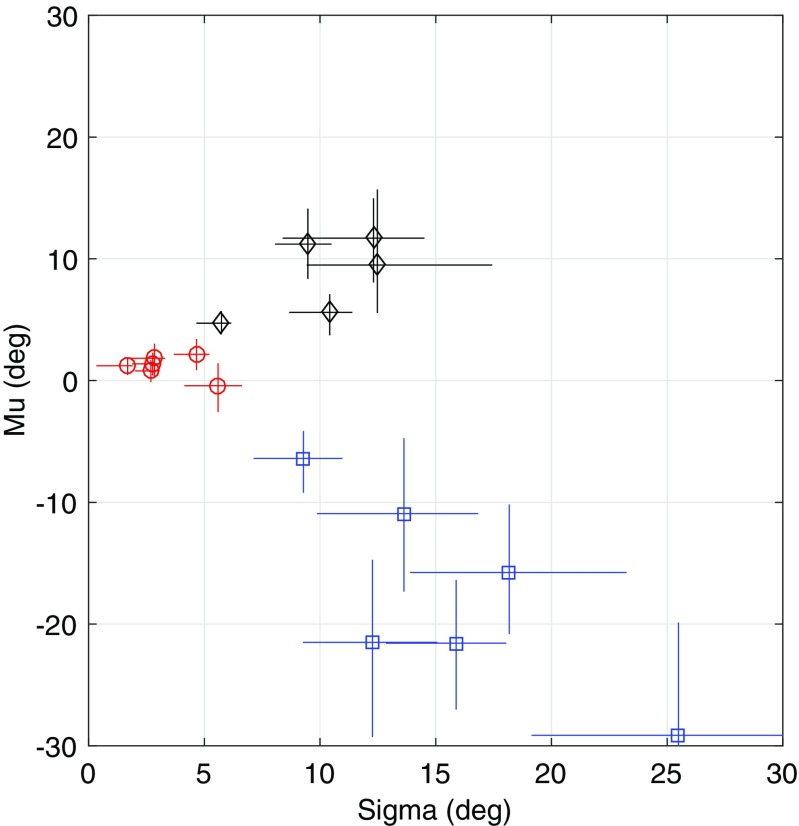
Results for Experiments 1-3. Each point represents a single subject in one of the experiments. The abscissa for each point is the estimate of internal noise. The ordinate is the estimate of perceptual offset away from zero obtained by the fit of the model described in the text. The error bars show 95% confidence intervals based on parametric bootstrapping. Results for Experiment 1 are shown in blue, for Experiment 2 in red, and Experiment 3 in black

To allow a more ready comparison between the results of the three experiments, Fig. 5 combines all the data into a single plot, in which each symbol represents the results for a single subject, with values of *μ* on the vertical axis and values of *σ* on the horizontal axis. The figure shows clear separation between the three experiments.

## Discussion

The repulsion of the trajectory of dots moving within an envelope, away from the envelope trajectory, is consistent with many previous findings on relative motion (Johannson, 1975, Dunkner, 1938, Cutting & Profitt, 1982; Dakin & Mareschal, 2000). Johannson presented an elliptically moving dot on an oscilloscope by applying sinusoidal input to the x and y amplifiers. By itself, the dot was seen as moving in an ellipse, but if it was flanked by horizontally-moving dots driven by the x signal the dot appeared to move vertically. We may say that it is only the component of the motion that is not accounted for by that of the flanks that is perceived. In the case of our dots and circular frame, the same analysis would run as follows. If the dots are moving vertically on the screen while the frame moves obliquely, they have a horizontal component to their motion *relative to the frame*, in the opposite direction to the horizontal component of the frame. They therefore are repulsed from the frame trajectory, as we find. A possibly simpler way of putting this is that dots moving vertically down the screen are actually moving horizontally within an obliquely moving frame, and this is what we see.

If the same effect operated in Experiment 2, where the dots moved obliquely and the frame vertically, the frame should have been repelled from the dots. That this did not happen is in accordance with Dunkner’s rule, that frames are more influential when they surround an object. The small effect we got was in the direction of attraction to the moving dot trajectory, in agreement with previous demonstrations of the “infinite regress” effect (Tse & Hsieh, 2006; Lisi & Cavanagh, 2015; Morgan, 2015). The fact that our effect was so small compared with previous demonstrations can be readily ascribed to (1) we used an explicit circular frame, providing a veridical first-order motion signal, as opposed to the second-order signal provided by a moving Gabor stimulus, and (2) the “infinite regress” effect is more pronounced in peripheral vision, while our stimulus passed through the fixation point. In Experiment 3, where we used parafoveal fixation and had no surrounding circle, the effect was larger, although still not as great in absolute terms as the repulsion in Experiment 1.

A recent Bayes-inspired analysis (Kwon et al., 2015) starts from the assumption that the actual trajectory of the frame or envelope is uncertain because of sensory noise particularly in the periphery, and the trajectory inferred by the observer is thus susceptible to alteration by movement of the texture within it. Putting this informally, if the moving dots are assumed to be a surface texture, one interpretation of their movement trajectory is that the object in which they are embedded is moving in that direction. Although expressed with greater mathematical precision, this account is not far from the Gestalt School proposition that our perception attempts to integrate the movement of multiple objects in the visual field in a physically plausible manner.

The finding that there can be both attraction and repulsion in similar figures echoes previous results on the effects of context with geometrical figures. The Muller-Lyer figure demonstrates attraction/assimilation of the longer line to the surrounding frame. It can be simplified to the “parallel lines” effect, where a line presented with two smaller, parallel flankers appears smaller than one surrounded by two larger flanks (Muller-Lyer, 1896). Yet in the Ebbinghaus figure, exactly the opposite is found with circles (Morgan, Melmoth & Solomon, 2013). It seems unlikely that a simple Kalman filtering process (Kwon et al., 2015) can account for both effects, at least not without a number of additional assumptions. The same holds for the “double drift” effects of context that we report.

To unify the repulsion found in Experiment 1 with the attraction found in Experiment 3, the following hypothesis is suggested. In agreement with Johansson and with Kwon et al., the dots are interpreted as the surface texture of a larger moving object. In the simplest case, the object and the dots have the same velocities, and the visual system assumes this to be the case unless there is contradictory sensory evidence. In Experiment 1, the dots move vertically and the envelope moves obliquely (30 deg). This is interpreted as the ball spinning around its vertical axis as it translates. The vertical retinal movement of the dots is resolved into a horizontal component due to spin and a vertical movement due to the object. If the subject is asked to report the perceived direction of the dots while ignoring the translation of the envelope they report the component due to spin. This is essentially the same as Johannson’s analysis of his elliptically moving dot stimulus.

In Experiment 3, the dots move obliquely and the envelope moves vertically. The key point is that without a frame the sensory evidence for the vertical motion is weak, just as it is for the second-order orientation of the envelope in the static Fraser “twisted cord.” The simplest interpretation of the stimulus is that the envelope is moving in the same direction as the dots, and this is only weakly contradicted by sensory evidence. If the sensory evidence for the envelope motion is made stronger by adding a frame, the frame is seen as moving more vertically (Experiment 2), and no doubt (as shown by Experiment 1) the dots are seen as spinning to account for the oblique component of their trajectory.
